# 

**Published:** 1885-10

**Authors:** 


					﻿Editorial.
Ohio State Dental Society.
The first annual meeting of this Society, since its re-organiza-
tion, will be held in Chillicothe on the last Wednesday of Oct.
inst., and every dentist who has any real interest and pride in
his profession in the State, ought to be present and do whatever
he can for the best interests of the society, and between this time
and the meeting, let every effort be put forth for its success.
Much has been done in Ohio for the dental profession in the
past that is gratifying to every true lover of his profession.
In Ohio, was organized, and is still in successful operation, the
.second oldest dental college in the world. This institution has
exercised an influence for good on the profession throughout this
country, and to some extent in other countries as well. In Ohio
has been edited and published the second oldest dental periodical
in the world. Of the influence of this everyone who has been in
the profession for ten years or more, knows much. Ohio was one
of the first to organize a state society; it was one of the first, if
not the first, to have enacted a law for the regulation of dental
practice, which required an examination and certificate of qualifi-
cation from those who proposed to enter its ranks. There have been
for many years within the state quite a number of local societies
some of them extending back about 40 years, others 15 to
20 years.
More has been done in Ohio in associative work than in any
other state in the union. Now in view of all these things consi-
derable effort must be put forth to sustain the reputation already
attained by the profession here; and it is hoped that every one
will bear these facts in mind and let them have their proper in-
fluence in stimulating to further effort. A great and pressing
need in the state now is for a revision of the law regulating den-
tal practice, though it was fully abreast and even in advance of
the times when it was enacted ; it has now become, in some re-
spects at least, superannuated, and needs revision. This is a work
requiring considerable effort and well directed wisdom. An ex-
cellent programme has been prepared and upon the subjects
there given, there will doubtless be a number of papers. We are
assured that at the coming meeting there will be quite a number
of visitors who will add great interest to the proceedings.
The programme has been sent to almost every dentist in the
state and need not here be given. The board of Examiners
meet at that time and we trust that all interested in this part of
the work will be present at the time indicated.
Harris’ Principles and Practice of Dentistry—11th
edition. Revised and edited by F. J. Gorgas, D.D.S., with
two full-page plates and 744 other illustrations. '8°; pp.
xxxix-994. Philadelphia: P. Blakeston, Son & Co. 1885.
The tenth edition of this excellent text-book appeared some
fourteen years ago. It was thoroughly revised and brought up
to the level of dental knowledge of 1871 by Dr. P. H. Ansten,
assisted by several co-editors. The edition now before us has re-
ceived the care of a no less able editor, and a comparison of the
two will enable us to summarize the changes in the practice
of dentistry during the period that has elapsed between 1871
and 1885.
In mere numerical respect the eleventh edition shows a gain
of 335 illustrations and 200 pages of text. In detail the new
edition includes a fair statement of the latest theories of the ori-
gin and formation of the teeth ; extended treatment of the sub-
jects, “Tumors of the Mouth,” “ Sensitiveness of Dentine,” and
“Malformed Teeth.” In part III a description is given of
contour fillings. The greatest changes, however, are observable
in part IV, devoted to mechanical dentistry. In the chapter on
metallo-plastic work, the space devoted to aluminum is reduced
from sixteen to three pages by the omission of the greater part
of the description of the method of Dr. Beau. In view of the
recent advances in the manufacture of this valuable metal, we
quote the following remarks, with wffiich we heartily agree:
“ The use of aluminum in dentistry is of recent origin, the
properties of the metal undeveloped, and its most appropri-
ate manipulations as yet undetermined. Although experiments
thus far indicate a want of durability, they reveal properties
which should stimulate to renewed effort in overcoming acknowl-
edged difficulties. Taking lesson from the injury which the
rcheapness and facility of vulcanite have inflicted upon prosthetic
dentistry, we may possibly find in aluminum a dental base pos-
sessed of an unsurpassed combinaton of excellencies; requiring
however, for their development an amount of time, care and skill,
that will exclude it from the practice of those who are doing such
discredit to their vocation.” It is to such work as is indicated
in the above paragraph that the sections of the American Dental
Association should apply the means recently appropriated to re-
search. .Let the dentist state clearly his requirements to a skilled
metallurgical enquirer and we may expect early and useful results.
The advance of Mechanical Dentistry is also shown in the
descriptions of the new bases Celluloid and Zylonite and in the
clear treatment of the subjects of continous gum and bridge and
graft work.
The ansesthetics at the command of the practitioner have re-
ceived the useful addition of hydrochlorate of cocaine, which how-
ever has hardly justified the extravagant expectations with which
this, as every other new substance, has been introduced.
We congratulate the publishers on the excellent manner in which
the work has been produced, paper, type and illustrations are all
equally good. We would, however, suggest that when a new
edition is published the names of Urbain Hemard, Raschkow and
Faveau, be correctly spelled.
Jhe Rental T^eqi^ter,
A Monthly Magazine Devoted to the Interests of the
DENTAL PROFESSION.
Terms Reduced to $2.00 Per Tear. Single Copies, 25 Cents.
EDITED BY PROF. J. TAFT, D.D.S.
-----AND------
Published by SA1H £ CROCKER R CO,, Ohio Dental and Surgical Depot.
The volume commences in January and closes in December.
The Register being issued monthly is always fresh, therefore Indispensable
to the practitioner who desires to keep posted up with the new inventions and
improvements which are daily developed. Neither expense nor effort will be
spared to give value and variety to its contents. Articles will be illustrated with
well executed engravings whenever they will add to the interest or assist to ex-
planation.
Its extensive eirculat:on and frequency of issue make it one of the BEST DEN-
TAL ADVERTISING MEDIUMS in the United States.
All subscriptions must begin with January or July.
Local or special notices, five lines or less, will be inserted for $3 each insertion ;
over five lines, 50 cts. per line.
All advertising bills payable in advance, or at furthest, after the issue of the
first number containing the advertisement. All transient advertisements to be
paid for in advance.
^CONTRIBUTIONS SOLICITED.
Exclusively Editorial Communications should be addressed to the Editor.
The latest number of the Register may be ordered with or without other goods
at any time, and all letters should be addressed to
SAM’L A. CROCKER & CO., Ohio Dental and Surgical Depot, Cincinnati, 0.
^HE 5ENTAL I^EQIpTER,
A Monthly Magazine Devoted to the Interests of the
DENTAL PROFESSION.
Terms Reduced to $2.00 Per Tear. Single Copies, 25 Cents.
EDITED BY PROF. J. TAFT, D.D.S,
------AND------
Publishes by SAK'D A, CROCKER & CO,, Ohio Denial and Surgical Eepsl,
The volume commences in January and doses in December.
The Register being issued monthly is always fresh, therefore Indispensable to the practitioner who desires to keep posted up with the new inventions and improvements which are daily developed. Neither expense nor effort will be spared to give value and variety to its contents. Articles will be illustrated with well executed engravings whenever they will add to the interest or assist to explanation.
Its extensive circulat:on and frequency of issue make it one of the BEST DENTAL ADVERTISING MEDIUMS in the United States.
All subscriptions must begin with January or July.
Local or special notices, five lines or less, will be inserted for 83 each insertion ; over five lines, 50 cts. per line.
All advertising bills payable in advance, or at furthest, after the issue of the first number containing the advertisement. All transient advertisements to be paid for in advance.
^CONTRIBUTIONS SOLICITED.
Exclusively Editorial Communications should he addressed to the Editor.
The latest number of the Register may be ordered with or without other goods at any time, and all letters should be addressed to
SAM’L A. CROCKER & CO., Ohio Dental and Surgical Depot, Cincinnati, 0.
				

